# More than just acral melanoma: the controversies of defining the disease

**DOI:** 10.1002/cjp2.233

**Published:** 2021-07-02

**Authors:** Sara S Bernardes, Ingrid Ferreira, David E Elder, Aretha B Nobre, Héctor Martínez‐Said, David J Adams, Carla Daniela Robles‐Espinoza, Patricia A Possik

**Affiliations:** ^1^ Program of Immunology and Tumour Biology Brazilian National Cancer Institute Rio de Janeiro Brazil; ^2^ Tissue Microenvironment Laboratory, Department of General Pathology Federal University of Minas Gerais Belo Horizonte Brazil; ^3^ Experimental Cancer Genetics Wellcome Sanger Institute Hinxton UK; ^4^ Université Libre de Bruxelles Brussels Belgium; ^5^ Division of Anatomic Pathology Hospital of the University of Pennsylvania Philadelphia PA USA; ^6^ Division of Pathology Brazilian National Cancer Institute Rio de Janeiro Brazil; ^7^ Serviço de Patologia, Maternidade Escola Universidade Federal do Rio de Janeiro Rio de Janeiro Brazil; ^8^ Servicio de Piel y Partes Blandas Instituto Nacional de Cancerología Ciudad de México Mexico; ^9^ Laboratorio Internacional de Investigación sobre el Genoma Humano Universidad Nacional Autónoma de México Santiago de Querétaro Mexico

**Keywords:** acral melanoma, epidemiology, histopathology, prognosis, genetics

## Abstract

Acral melanoma (AM) is a malignant cutaneous melanocytic tumour specifically located on the palms, soles, and nail apparatus, which are areas of glabrous (hairless) skin. Acral lentiginous melanoma, a subtype of AM, represents a histopathological subtype diagnosis of cutaneous melanoma with unique morphological and structural features. Despite clear definitions, the misuse of these terms and the inconsistency in reporting the histopathological features of AM cases have become a major obstacle to the study of the disease. In this review, we discuss the epidemiology, histopathological features, prognosis, and genetic profile of AM, highlighting the differences observed when histopathological subtypes are considered. The increasing global effort to characterise AM cases from ethnically diverse populations would benefit greatly from a more consistent classification of the disease.

## Introduction

The terms used to denote melanomas from volar surfaces of hands and feet or the nail apparatus, ‘acral melanoma’ and ‘acral lentiginous melanoma’, embody distinct diagnoses. According to the World Health Organization (WHO), acral melanoma (AM) is an anatomical term that refers to melanoma located on glabrous (hairless) skin of the extremities. On the other hand, acral lentiginous melanoma (ALM) represents a histopathological subtype of cutaneous melanoma with characteristic morphological and structural features, recognised alongside others such as lentigo maligna melanoma (LMM), superficial spreading melanoma (SSM), and nodular melanoma (NM) [1].

Almost two decades ago, attention was drawn to the various controversial aspects and unanswered questions about AM, including the misuse of the terms AM and ALM [2]. Apparently, the lack of consensus on how to refer to melanoma in acral skin continues until today: from all original studies retrieved from a search of PubMed using ‘acral melanoma’ as the search term, only 38% specified the histopathological subtype of the lesions, 78% reported the anatomical sites, and 37% reported information on both (Table 1). In 21% of the studies, no specification of the histopathological subtype and anatomical site was reported. The search does not retrieve all studies on AM, but it offers us a picture of what is available in the literature. These studies involve different populations and illustrate the lack of reporting rigour and consensus.

**Table 1:** Articles focusing on AM according to the histopathological subtype and anatomical site.

Publication year	Histopathological subtypes	Anatomical site	Reference
2020	ALM	Sole, palm, nail apparatus	[3]
2020	Not specified	Acral non‐nail, nail apparatus*	[4]
2020	Not specified	Sole, toe, finger, nail apparatus*	[5]
2020	Not specified	Hand, foot, finger, toe, sole, nail apparatus*	[6]
2020	Not specified	Sole, palm, nail apparatus	[7]
2020	Not specified	Not specified	[8]
2020	ALM, NM	Sole, palm, nail apparatus	[9]
2020	ALM, NM, SSM	Hand, foot, nail apparatus*	[10]
2020	ALM, NM, SSM, LMM	Hand, foot*	[11]
2020	Not specified	Not specified	[12]
2020	Not specified	Not specified	[13]
2020	Not specified	Not specified	[14]
2019	ALM	Sole, palm, nail apparatus	[15]
2019	ALM, NM, SSM, LMM	Not specified	[16]
2019	Not specified	Hand, foot*	[17]
2019	Not specified	Not specified	[18]
2019	ALM, NM, SSM	Sole, palm, dorsum of hand and foot, nail apparatus	[19]
2019	Not specified	Not specified	[20]
2019	ALM	Sole, lateral great toe	[21]
2019	ALM, NM, SSM	Sole, palm, nail apparatus	[22]
2019	Not specified	Not specified	[23]
2019	Not specified	Sole	[24]
2019	Not specified	Sole, palm, nail apparatus	[25]
2018	Not specified	Sole, palm, nail apparatus, digit	[26]
2018	Not specified	Hand, foot*	[27]
2018	ALM, NM, SSM	Sole, palm, nail apparatus	[28]
2018	Not specified	Nail apparatus, non‐nail apparatus*	[29]
2018	Not specified	Sole, palm	[30]
2018	Not specified	Not specified	[31]
2018	ALM, SSM, NM	Sole, palm, nail apparatus	[32]
2018	Not specified	Not specified	[33]
2018	Not specified	Hand, foot*	[34]
2018	Not specified	Hand, finger, foot and toe*	[35]
2018	Not specified	Hand, foot, sole, finger*	[36]
2018	ALM, NM, SSM	Palm, sole, nail apparatus	[37]
2018	ALM, NM, SSM, LMM	Sole, palm, dorsum of hand and foot, nail apparatus, interdigital	[38]
2017	Not specified	Not specified	[39]
2017	Not specified	Not specified	[40]
2017	Not specified	Foot, palm, nail apparatus*	[41]
2017	ALM	Sole, nail apparatus	[42]
2017	Not specified	Foot, hand, nail apparatus*	[43]
2017	ALM	Sole, palm, nail apparatus	[44]
2016	Not specified	Hand, foot, nail apparatus*	[45]
2016	Not specified	Sole, toe, top of the foot, palm, finger, near the fingernail*	[46]
2016	ALM, NM	Sole, palm, dorsum of hand and foot, nail apparatus	[47]
2016	ALM, NM, SSM	Sole, toe, dorsum of the foot, finger, nail apparatus	[48]
2016	ALM, non‐ALM	Sole, palm, nail apparatus	[49]
2016	ALM, NM, SSM	Sole, palm, nail apparatus	[50]
2016	ALM, non‐ALM	Sole, palm, nail apparatus	[51]
2015	Not specified	Sole, palm	[52]
2015	Not specified	Not specified	[53]
2015	Not specified	Sole, palm, nail apparatus	[54]
2015	Not specified	Hand, foot*	[55]
2014	Not specified	Foot, sole, toe*	[56]
2013	Not specified	Not specified	[57]
2013	ALM, NM, SSM	Sole, palm, nail apparatus	[58]
2013	ALM, NM	Sole, palm, nail apparatus	[59]
2013	Not specified	Sole, palm, nail apparatus	[60]
2013	ALM, NM, desmoplastic	Sole, palm, nail apparatus	[61]
2013	Not specified	Sole, palm, dorsum of hand and foot	[62]
2013	ALM, NM	Nail apparatus	[63]
2012	Not specified	Sole, nail apparatus, web space	[64]
2012	Not specified	Sole, toe, web space	[65]
2012	Not specified	Hand, foot*	[66]
2011	Not specified	Not specified	[67]
2011	Not specified	Not specified	[68]
2011	Not specified	Not specified	[69]
2009	Not specified	Not specified	[70]
2005	ALM	Sole, palm, finger, toe, nail apparatus*	[71]
2004	Not specified	Sole, palm, nail apparatus	[72]
2004	ALM	Sole, palm	[73]
2000	ALM, NM, SSM	Sole, palm, nail apparatus, dorsum of hand, foot	[74]
2000	ALM, SSM	Sole, toe, foot*	[75]
1998	ALM, NM, SSM	Sole, palm, nail apparatus	[76]
1993	Not specified	Sole, palm, nail apparatus, dorsum of hand and foot	[77]
1990	ALM, NM, SSM	Sole, palm, nail apparatus	[78]
1990	Not specified	Sole, palm, nail apparatus	[79]
1988	Not specified	Sole, palm, nail apparatus	[80]
1985	ALM, NM, SSM	Sole, palm, nail apparatus	[81]
1983	Not specified	Sole, palm, nail apparatus, toe, finger, dorsum of foot	[82]
1982	Not specified	Sole, palm, dorsum of hand and foot	[83]

A search using the term ‘acral melanoma’ on PubMed in June 2020 (English language and journal article type) retrieved 239 articles (published and ahead of print), of which 154 were original studies. Of those, 94 used the words ‘acral’ and ‘melanoma’ in the title. Four were excluded for using the word ‘lentiginous’ in the title and nine were excluded for other reasons (file not available, only *in situ* AM, naevi, or cell lines), resulting in 81 articles. Among these articles, 38% reported the histopathological subtypes studied, 78% reported the anatomical sites, and 37% reported information on both. In 21% of the studies, there was no specification of the histopathological subtype and anatomical site.

Not specified: information on histopathological subtype or anatomical location of AM cases was not provided. The volar surfaces of the fingers and toes are included as palms and soles, respectively. Specific locations of the soles, such as the heels, are included as soles.

* Articles in which it is unclear whether the classification was performed according to the 2018 WHO guidelines.

These inconsistencies regarding the reporting of histopathological subtypes have a major impact in the interpretation of data derived from studies aimed at understanding the epidemiological, clinical, and biological characteristics of AM. In the following sections, we discuss different aspects of AM, highlighting the differences observed when the histopathological subtypes are considered, and in doing so we aim to draw attention to the importance of an accurate anatomical and histopathological classification. As an important note, we clarify that we only reviewed articles that followed the WHO guidelines for diagnosing AM [1].

## AM epidemiology

According to several population‐based epidemiology studies, AM represents only about 3% of melanomas that occur in European‐descent individuals [84, 85, 86], whereas it represents a higher proportion of cases in Asian, Hispanic, and African populations [1]. For example, AM accounts for more than 40% of cutaneous melanoma in Asia [87, 88] and 20.1% in Latin American countries [89]. Furthermore, the relative frequency of AM is higher in genetically heterogeneous or Amerindian‐descent populations (indigenous Latin Americans) than in European‐descent individuals from Latin American countries [90, 91].

In the case of ALM, its absolute global incidence is similar across populations and is much lower than that of SSM or LMM. However, it also accounts for a higher percentage of total melanoma cases in non‐European descent populations compared to populations of European descent [92].

These contrasts in the relative incidence of AM and ALM may be ascribed to marked differences in sun‐induced melanoma incidence among these populations; for example, the incidence of cutaneous melanoma is 49 per 100,000 individuals in Australia, which has a predominantly European‐descent population [93], but is typically estimated to be less than 5 per 100,000 inhabitants in several countries in Latin America [91, 94, 95]. As AM and ALM have similar incidences around the globe and are not thought to be ultraviolet (UV)‐related, they constitute a higher proportion of cases in countries with a lower incidence of melanoma.

Among studies that have reported the histopathological subtypes of AM, a high relative frequency of ALM is consistently reported, but the frequencies of AM histopathological subtypes vary considerably. ALM has been reported to represent 44–90% of AM, while acral NM (2.5–41%) and acral SSM (0.7–28%) are relatively less frequent subtypes of AM [16, 22, 28, 38, 50, 58, 76, 96]. When studying specific populations, these variations are noteworthy. In European‐descent individuals, ALM represents ~72% of AM [16, 38, 76], followed by acral SSM (~18%) [16, 38, 76]. In Asians, ~88% of AM patients were reported as ALM, whereas the second most frequent subtype was NM, with ~11% of cases [50, 58]. In African individuals, ALM corresponds to ~78% of AM [96]. In Brazil, which consists of a highly genetically heterogeneous population with African, European, and indigenous heritage, ALM (56%) and acral NM (22%) represent the majority of cases, whereas acral SSM represents only 13% of AM cases [22, 28].

While this variation is interesting, it requires special attention because, although it may indicate real differences among populations, it may also indicate a lack of consensus in the classification of these types of tumours. It also shows how important it is to perform studies considering the diversity of the world population in order to obtain a more accurate picture of the characteristics of this disease.

## The distinct clinicopathology of AM

According to the 2018 WHO classification of melanoma, AM is grouped together with melanomas not consistently associated with cumulative solar damage. In fact, because of the anatomical location and the thick stratum corneum of glabrous skin, UV radiation is not believed to play a significant role in the pathogenesis of AM [97]. Since 1935, trauma has been suspected to influence the development of melanoma on the soles of the feet (Hewer, as cited in Ref. [98]). A strong association has yet to be proven, but several studies have shown a higher incidence of plantar melanoma in areas of greater physical stress such as the heels [58, 99, 100].

Clinically, AM is usually characterised by an asymmetric pigmented macule with highly irregular notched borders and various colour shades (ranging from tan to dark brown to black) in the centre of which a nodule may develop. In subungual locations, melanoma presents as longitudinal or total (damage of the entire nail plate) melanonychia. Periungual pigmentation (Hutchinson sign) and nail plate dystrophy are also clues to a diagnosis of nail apparatus melanoma (or subungual melanoma) [101].

**Figure 1:**
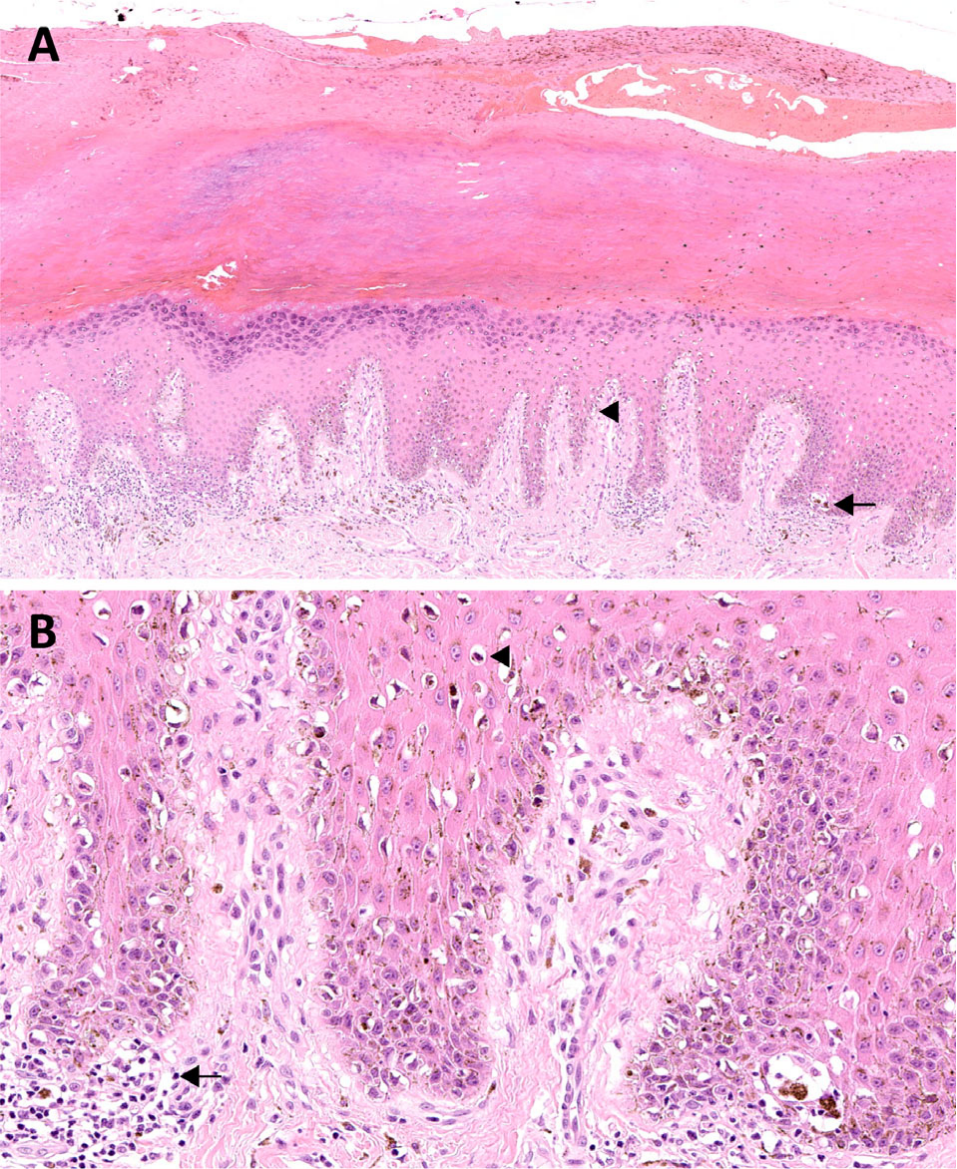
Acral melanoma. (A) *In situ* acral lentiginous melanoma with a lentiginous proliferation of atypical melanocytes along the dermoepidermal junction (arrowhead), with pagetoid spread and scattered nests at the tips of the rete ridges (arrow). There is also acanthosis, hypergranulosis, and hyperkeratosis (H&E, ×100). (B) Tumour cells are hyperchromatic and have a cytoplasmic fixation retraction artefact (arrowhead). The dermis contains a chronic inflammatory cell infiltrate associated with macrophages (arrow) (H&E, ×400).

**Figure 2:**
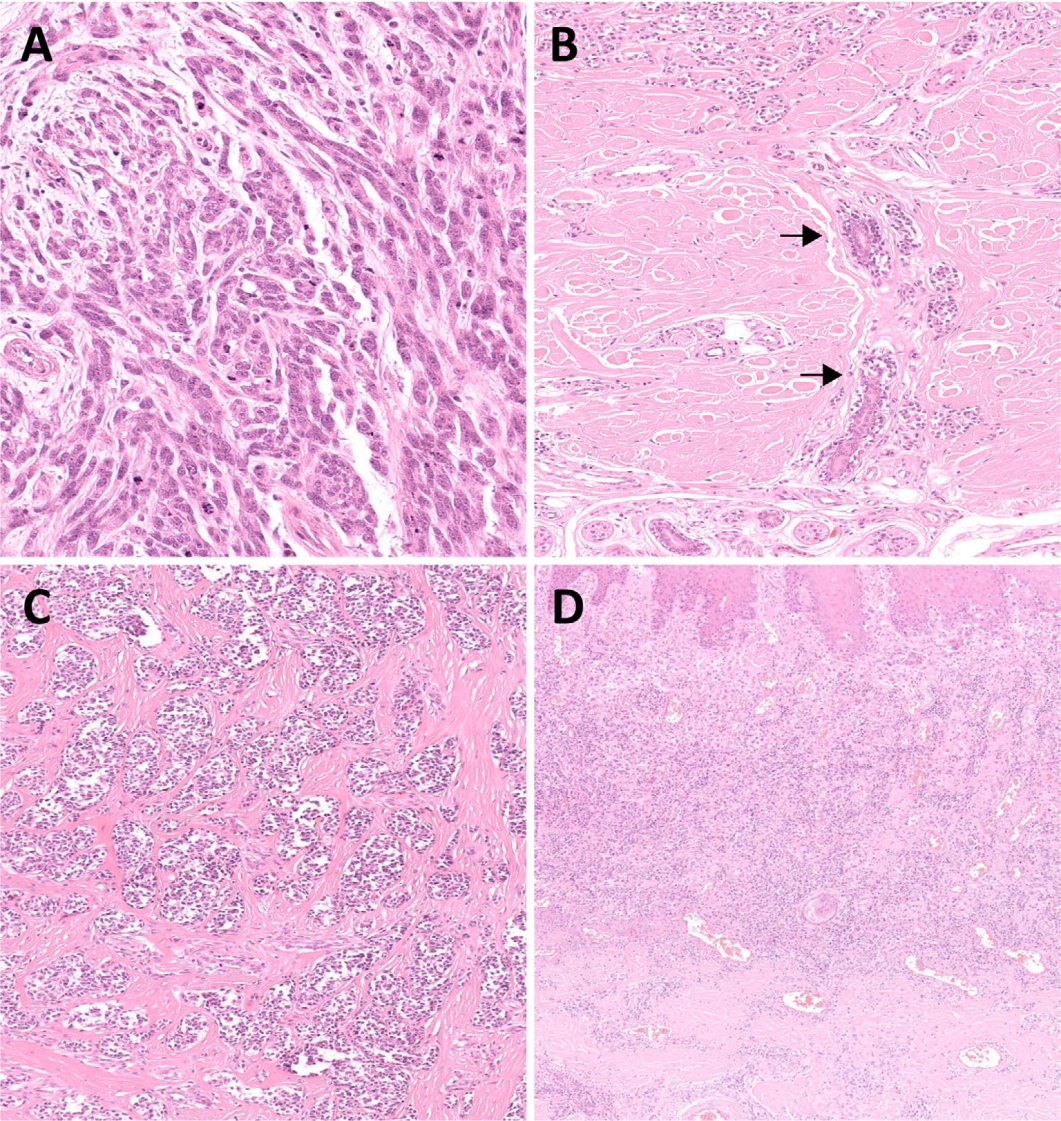
Acral lentiginous melanoma. (A) Tumour cells are usually spindle. (B) Deep extension along sweat gland (arrows) is frequent. Desmoplastic reaction (C) and band‐like chronic inflammatory cell infiltrate (D) are associated features (H&E, ×400, ×200, ×200, and ×100).

Histopathologically, ALM is characterised by a lentiginous growth pattern, i.e. a continuous proliferation of atypical melanocytes with enlarged hyperchromatic nuclei and prominent dendrites along the dermoepidermal junction. The confluence of the cells increases with the stage of the lesion varying from scattered cells along the basal layer of an epidermal hyperplasia at early stage to pagetoid spread and junctional nests at late stage, the latter usually at the tips of the epidermal ridges or haphazardly oriented. Established lesions show acanthosis with marked elongation of the rete ridges (Figure 1). Cells are usually spindle (Figure 2A) and pigmented. However, an epithelioid component is often present in invasive lesions and can be predominant. Giant cells can also be seen. Extension along sweat glands is commonly observed (syringotropism) (Figure 2B). Invasive tumours can display a desmoplastic reaction (Figure 2C) and neurotropism. A band‐like chronic inflammatory cell infiltrate can surround the tumours (Figure 2D) [1, 101, 102].

**Figure 3:**
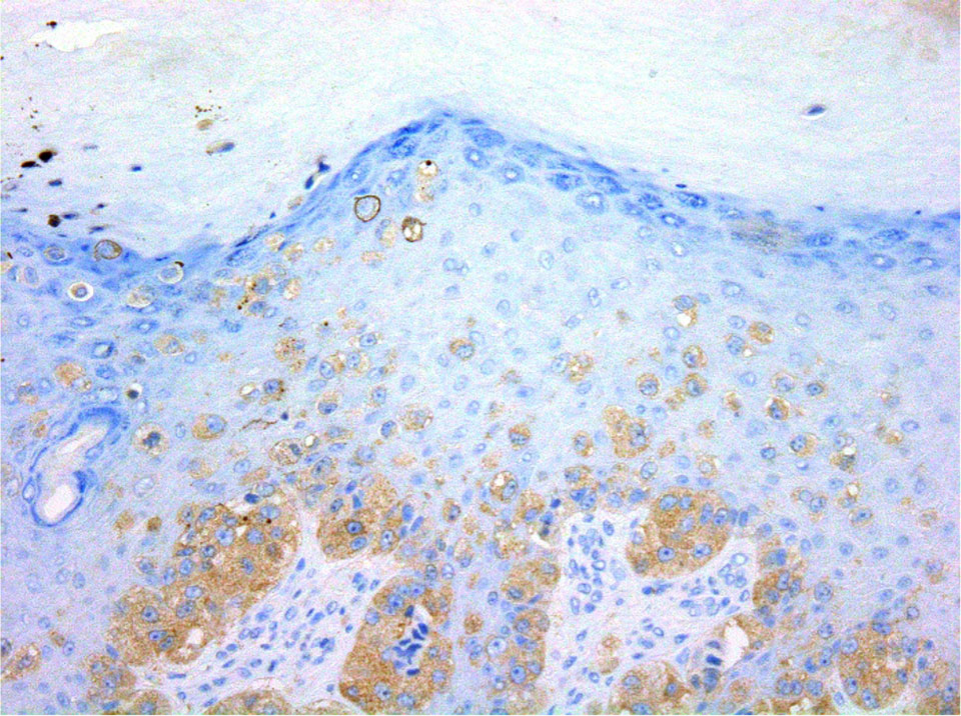
Superficial spreading melanoma in acral skin. Because of the upward spread of abnormal melanocytic cells in the epidermis, it may also be referred to as acral pagetoid melanoma. Lesion stained positively for the BRAF^V600E^ protein (BRAF^V600E^/DAB, ×400).

**Figure 4:**
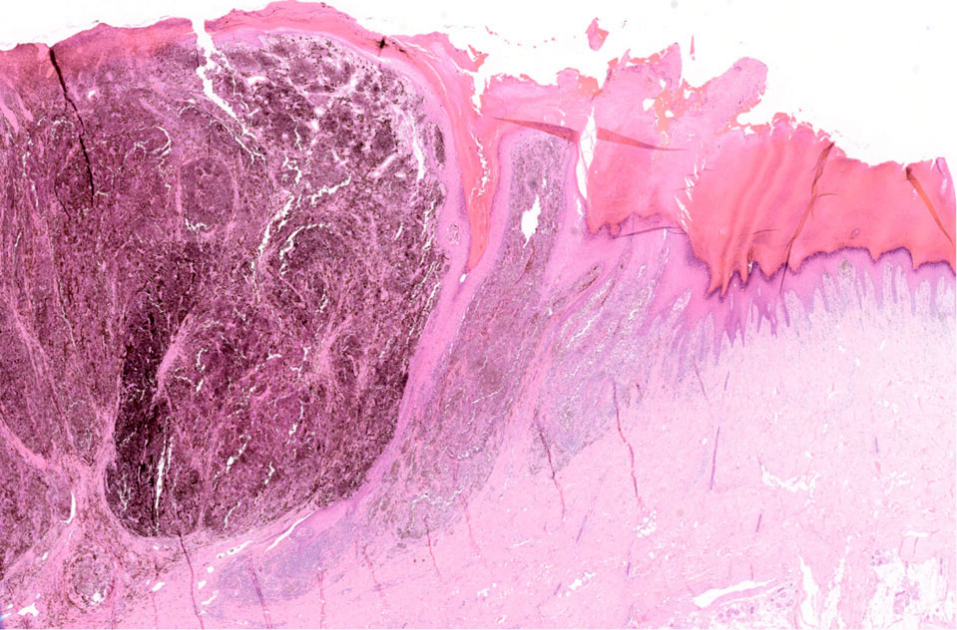
Acral lentiginous melanoma with a ‘nodular‐like’ vertical growth phase (on the left). There also is an adjacent radial growth phase including a lentiginous proliferation of atypical melanocytes along the dermoepidermal junction (on the right) (H&E, ×20).

Distinguishing ALM from SSM can sometimes be challenging. Compared to ALM, SSM is mainly characterised by a nested and pagetoid proliferation of epithelioid melanocytes along the dermoepidermal junction (Figure 3). However, it is not entirely specific as both of these features can also be observed in advanced ALM. Changes at the periphery of the lesions may be more relevant for classification. Importantly, SSM is mainly encountered on the dorsa of hands and feet, which, according to the WHO, are not considered acral sites [97]. Although the prevalence of *KIT* mutations is low in ALM (~15%) [103], the presence of KIT expression can be useful in complex diagnostic cases. By immunohistochemistry, in general (subject to exception [104]), KIT expression is in favour of ALM [103] as *KIT* mutations appear to be related to the lentiginous growth pattern [105], while the expression of BRAF^V600E^ argues for non‐ALM/SSM subtype [37, 106]. These two histopathological subtypes can present with a prominent vertical growth phase, which can be misdiagnosed for an NM. They are distinguished from NM by the presence of an adjacent radial growth phase [1, 102, 107, 108] (Figure 4).

Similar to non‐acral cutaneous melanomas, histopathological features such as Breslow depth, ulceration, and sentinel lymph node (SLN) status have been identified as prognostic factors in AM [22, 28, 60, 109, 110]. In AM, Breslow depth is usually high and is considered an independent prognostic factor and positively correlates with ulceration [28]. Consistently, in amelanotic AM, in which acral NM was the most common histopathological subtype (63.6%), ulceration was observed in 83% of cases, and more than 50% of cases had Breslow depth over 4 mm [59]. Also, it has been observed that ulceration is an important predictive factor for SLN involvement in ALM [111]. Thus, although the importance of these histopathological features is well established in AM, studies that look at the different subtypes of AM in order to better understand the impact of these characteristics on patient prognosis are still needed.

## The molecular profile of AM

The consensus that AMs are molecularly different from cutaneous melanomas of non‐glabrous skin is well accepted in the literature. AM displays a lower frequency of *BRAF* ^V600E^ mutations than non‐acral cutaneous melanoma but achieves activation of the MAPK pathway through alterations in *NRAS*, *NF1*, *KIT*, and other genes such as *SPRED1*, a negative regulator of RAS [25, 112, 113, 114, 115]. Genomic aberrations in *KIT*, *CCND1*, *CDK4, GAB2*, and *TERT* are common [25, 114, 116, 117], but what best describes the genomic landscape of AM is a lower number of point somatic mutations and an increased frequency of genomic aberrations [112, 113, 114, 115].

Most studies, however, were based on heterogeneous cohorts that pooled together several distinct AM subtypes and, in most cases, without discriminating tumours according to the WHO histopathological classification (Table 1). Although these studies mostly agree on the general molecular features of AM, the lack of information regarding histopathological parameters prevents the investigation of potential differences among AM subtypes.

## Unanswered questions and perspectives

Herein, we have highlighted different aspects of AM drawing attention to the lack of information on the histopathological classification of the tumours.

Our review of the literature revealed that only 38% of the studies that included AM cases reported histopathological subtype information. Because most studies of AM do not classify samples by their histopathological subtype, it is difficult to evaluate whether the different aspects of the disease – e.g. incidence, prognosis, and molecular landscape – apply to all patients with AM or just to patients with a specific histopathological subtype such as ALM.

There are other concerns not discussed here in great depth that deserve major attention. One is related to the inconsistency in diagnosing AM according to the type of skin in which it occurs. Several studies consider melanomas arising at the dorsal surface of the hands and feet as AMs, disregarding the WHO guidelines that recommend that AMs always occur on glabrous acral skin [1]. Not surprisingly, whereas 78% of the studies retrieved in our literature search on AM reported anatomical localisation of the lesions, only half of these confirmed their location on glabrous acral skin (Table 1). This is worrisome because studies that compared melanomas involving dorsal surfaces of hands and feet, which are more frequently classified as SSM, to melanomas on glabrous acral skin reported significant differences, including in the genetics of these tumours and in patient prognosis [38, 89, 91].

Another relevant point of discussion is how similar subungual melanomas are to AMs occurring on the palms and soles. Recent evidence shows significant clinical and molecular differences that may impact diagnosis and treatment [118]. Among them, reported trauma and *KIT* mutations have been more frequently associated with subungual melanomas than with AMs occurring in other locations. Whether these differences are sufficient to categorise subungual melanomas as a separate entity still needs to be discussed, but findings like these demonstrate both the advantage of considering different groupings when studying AM and how this can help arrive at optimal definitions.

In conclusion, AM is a cutaneous melanoma subtype which exhibits its own clinical and genetic features. Nevertheless, the inconsistency regarding the definition used for AM and the lack of histopathological information observed in many scientific articles limits our knowledge of its biology. In the future, greater attention should be given to the definition and classification of AMs to clarify the extent to which different histopathological subtypes do occur on acral sites across different ethnic populations. This information will be key to helping characterise the biological, clinical, and molecular aspects of AM and its histopathological subtypes. All these studies will hopefully contribute to decreasing the gaps in knowledge of this disease and advance its therapeutic possibilities.

## Author contributions statement

SSB, CDR‐E, DJA and PAP conceived and designed the review. SSB performed the bibliographical search. IF, DEE and SSB designed the figures. All authors participated in writing and approved the final version of the manuscript.
